# Understanding Depression in Autism: The Role of Subjective Perception and Anterior Cingulate Cortex Volume

**DOI:** 10.21203/rs.3.rs-4947599/v1

**Published:** 2024-09-20

**Authors:** Yu Hao, Sarah Banker, Jadyn Trayvick, Sarah Barkley, Arabella Peters, Abigael Thinakaran, Christopher McLaughlin, Xiaosi Gu, Jennifer Foss-Feig, Daniela Schiller

**Affiliations:** 1. Nash Family Department of Neuroscience, Icahn School of Medicine at Mount Sinai, New York, NY, USA; 2. Department of Psychiatry, Icahn School of Medicine at Mount Sinai, New York, NY, USA; 3. Seaver Autism Center for Research and Treatment, Icahn School of Medicine at Mount Sinai, New York, NY, USA; 4. Center for Computational Psychiatry, Icahn School of Medicine at Mount Sinai, New York, NY, USA

**Keywords:** autism spectrum disorder, depression, anterior cingulate cortex, amygdala, social impairment, self-awareness, affiliation

## Abstract

**Background:**

The prevalence of depression is elevated in individuals with autism spectrum disorder (ASD) compared to the general population, yet the reasons for this disparity remain unclear. While social deficits central to ASD may contribute to depression, it is uncertain whether social interaction behavior themselves or individuals’ introspection about their social behaviors are more impactful. Although the anterior cingulate cortex (ACC) and amygdala are frequently implicated in ASD, depression, and social functioning, it is unknown if these regions explain differences between ASD adults with and without co-occurring depression.

**Methods:**

The present study contrasted observed vs. subjective perception of autism symptoms and social performances assessed with both standardized measures and a lab task, in 65 sex-balanced (52.24% male) autistic young adults. We also quantified ACC and amygdala volume with 7-Tesla structural neuroimaging to examine correlations with depression and social functioning.

**Results:**

We found that ASD individuals with depression exhibited differences in subjective evaluations including heightened self-awareness of ASD symptoms, lower subjective satisfaction with social relations, and less perceived affiliation during the social interaction task, yet no differences in corresponding observed measures, compared to those without depression. Larger ACC volume was related to depression, greater self-awareness of ASD symptoms, and worse subjective satisfaction with social interactions. In contrast, amygdala volume, despite its association with clinician-rated ASD symptoms, was not related to depression.

**Limitations:**

Due to the cross-sectional nature of our study, we cannot determine the directionality of the observed relationships. Additionally, we included only individuals with an IQ over 60 to ensure participants could complete the social task, which excluded many on the autism spectrum. We also utilized self-reported depression indices instead of clinically diagnosed depression, which may limit the comprehensiveness of the findings.

**Conclusions:**

Our approach highlights the unique role of subjective perception of autism symptoms and social interactions, beyond the observable manifestation of social interaction in ASD, in contributing to depression, with the ACC playing a crucial role. These findings imply possible heterogeneity of ASD concerning co-occurring depression. Using neuroimaging, we were able to demarcate depressive phenotypes co-occurring alongside autistic phenotypes.

## BACKGROUND

Depression is one of the most commonly observed co-occurring psychiatric conditions in adults with autism spectrum disorder (ASD) [1], yet the reasons behind this co-occurrence remain unclear. The lifetime prevalence of depression in ASD is estimated to be 10%−49% depending on the measurement, which is significantly higher than rates in the general population [2–4]. The presence of comorbid depression may substantially impact quality of life for adults with ASD, affecting their education, employment, and satisfaction with social relationships [5–7]. Understanding the phenotypes that differentiate depression and ASD could point toward new intervention paths to improve psychosocial outcomes, adaptive functioning, and overall quality of life for adults with ASD.

Depression is in general linked to social factors such as the number, quality, and reciprocity of social relationships [8]. Given that interpersonal difficulties are central to an ASD diagnosis [9], there is compelling evidence to suggest that social factors play a critical role in the etiology of depression in adults with ASD [10,11]. However, there are mixed findings on the relationship between clinician-rated ASD symptom severity and depression [12–16] Additionally, there may be significant variability among adults with ASD in their awareness of their social interaction successes or difficulties. Young people with ASD who are more conscious of themselves, and their social difficulties tend to experience greater emotional pain when faced with social failures, the risk of which increases when social interactions are more conflictive [17,18]. While social deficits in ASD may contribute to depression, it remains unclear whether it is the behaviors during social interactions themselves or the awareness of difficulties in these interactions and subsequent feelings about social connections that have a greater impact on depression in ASD individuals.

Findings on the relationship between self-reported autism symptoms and depression in young adults with ASD are limited. For example, Gotham et al. (2014) found that greater self-perceived autistic impairments in young adults were associated with increased depression symptoms [19], while Murray et al. (2019) found no such relationship [20]. Other measures of self-perception of social interaction, such as perceived lack of tangible social support, feeling different from others, and low self-perceived social competence, have been associated with increased symptoms of depression with ASD [21–23]. Additionally, Day et al. (2019) found that higher self-awareness of social impairments, defined as being aware of one’s disability and differences from others, predicted depression symptoms among young adults with ASD who had average or above average IQ [24]. This evidence suggests the need for more research to differentiate between observable measures, such as clinician-rated autism symptoms, and subjective perceptions of autism symptoms and social difficulties in the development of depression.

The present study aimed to understand the contributions of observed vs subjective perception of autism symptoms and social performance on the occurrence of depression in ASD. To examine the effect of autism symptoms on depression, we considered both clinician-assessed autism symptoms and participants’ self-rated autism symptoms. To examine social interactions, we first incorporated surveys to assess frequency of social contacts, subjective satisfaction with social relations, and then we examined social interaction effects on depression. Network size in social contacts was related to depression [25], but subjective satisfaction with social interactions might matters more for quality of life in the general population [26]. If self-awareness and introspection are important contributors to depression in ASD, we predicted that worse subjective satisfaction in social relations would have a greater impact on depression in the ASD population. We next utilized an innovative social interaction task designed to dynamically probe social affiliation during interactions with virtual characters. Past research suggests that individuals with ASD show lower social affiliation (sharing personal information or physical contacts) than typically developing (TD) individuals [27]. We used task-based affiliation behavior and perceived affiliation to predict depression in ASD. These paired examinations of observed versus subjective indices of autism symptoms and social interaction traits can help us understand the role of ASD-related social impairment in the etiology of depression, and why autistic individuals are more prone to depression than others.

The other main aim of the present study was to understand the contributions of brain structure on the occurrence of depression in ASD. There is evidence of structural brain differences both in autism and in depression, but limited research about how these differences may intersect in autism with co-occurring depression. We conducted a priori tests of brain regions previously implicated in both the ASD and/or depression literatures: the anterior cingulate cortex (ACC) and amygdala. We investigated whether the volumes of these brain regions in autistic individuals are associated with their ASD symptoms, social behaviors and perceptions, and the presence of depression. Anatomical and functional evidence suggests that the ACC contributes to social cognition by estimating the motivation of others and dynamically updating these estimates when new evidence suggests they were incorrect (see review from Apps et al. 2016 [28]). Studies have found functional abnormalities in the ACC in individuals with ASD, either through resting-state fMRI [29] or task-based fMRI [30–33] investigating social deficits, and thinner cingulate in ASD compared with typically developed individuals [34,35]. Less is known about how the volume of the ACC relates to ASD-related phenotypes. ACC volumetric reduction is considered a biomarker in major depressive disorder and self-rated depressive symptom severity [36–39], but its relationship to depression in ASD is not well understood. Moreover, cumulative evidence has demonstrated abnormalities in the amygdala structure and its function during social and emotional processing in autism [40–42] and a correlation between amygdala volumes and autism symptoms severity [43–48]. As the amygdala volume has frequently been studied in association with anxiety in ASD [49,50], it can be a region of interest to contrast with the ACC to better understand the relationship between ASD phenotypes and depressive phenotypes in ASD.

Our study utilized 7T structural neuroimaging on a sample of adults with ASD with and without depression. We investigated ACC and amygdala volume in relation to ASD symptoms, depression and social behaviors, which can help us demarcate the biological phenotypes of co-occurring depression from those of ASD and examine their relation to behavioral phenotypes.

## METHODS

### Participants

Participants were recruited through our local research registry, physical flyers around New York City, email listserv announcements, and word of mouth. The eligibility criteria were: (1) ages 18 to 50, (2) meeting DSM-5 criteria for ASD, (3) having an IQ over 60 (assessed by the Wechsler Abbreviated Scale of Intelligence and Wechsler Intelligence Scale for Adults), (4) having no history of neurological concerns like epilepsy or traumatic brain injury, and (5) no substance or alcohol abuse disorders nor recreational drug use. ASD screening was conducted by licensed clinicians using the ADOS-2, supplemented with developmental and clinical history as needed, to inform DSM-5 criteria.

Since there is no prior research on the relationship between brain structure and depression in ASD, and no studies examining the contrast between objective and subjective evaluations of social functioning, we couldn’t perform a power analysis to estimate the sample size. We utilized a sample collected from a grant investigating social functioning in ASD (Grant No. R01MH122611; Foss-Feig, Gu, Schiller). In total, 104 autistic individuals participated in the study and 92 participants had complete demographic and IQ measures. Among these 92 participants, 73 completed the social task and 72 underwent MRI scan. Seven participants’ MRI data was removed due to bad quality, and the final analysis included 65 sex-balanced (52.24% male) high-functioning young adults with ASD (mean age = 27.32, SD = 8.77) for volumetric analysis. The Icahn School of Medicine at Mount Sinai’s Institutional Review Board approved the study protocol. All participants gave written informed consent and received compensation for their participation.

### Clinical assessment of autism and depression

Clinical assessments of autism symptom severity and intelligence were collected in addition to self-rated autism symptom severity, self-rated depression symptoms and self-reported depression diagnoses. Demographic information and statistics of clinical assessment is shown in Table 1.

#### ADOS-2 module 4

The Autism Diagnostic Observation Schedule, 2nd edition (ADOS-2 [51]), is a semi-structured diagnostic tool for autism that includes different modules for varying levels of verbal and developmental abilities. Module 4 was used in this study, which was comprised fully of verbally fluent adults. In the following sections, we will refer to this as *clinician-rated ASD symptoms*.

#### Broader Autism Phenotype Questionnaire

The Broader Autism Phenotype Questionnaire (BAPQ) is a self-report questionnaire for adults that contains 36 statements. It yields subscales measuring aloofness, rigidity, and pragmatic language. This questionnaire has succeeded in meeting both sensitivity and specificity requirements for detecting the broader autism phenotype [52]. Its robust psychometric properties [52,53]and absence of ceiling effects [54] in individuals with ASD indicate that it performs effectively in both clinical populations [55] and the general population. In the following sections, we will refer to this as *self-rated ASD symptoms*.

#### Intelligence Quotient

The Wechsler Abbreviated Scale of Intelligence, Second Edition (WASI-II), is a measure of cognitive ability for individuals aged 6–90 years. The Full Scale IQ (FSIQ) was calculated based on the administration of all four subtests (FSIQ-4 [56]). In addition, the Wechsler Intelligence Scale for Adults – Fourth edition (WAIS-IV [57]) is used to assess intellectual profile for people between 16 and 90 years old. It is composed of four scores and a general intelligence index. The four indexes are VCI, PRI, WMI and PSI. In our sample, 16 participants completed the WAIS and 81 participants completed the WASI. In the following sections, we will refer to these scales as *IQ*.

#### Self-reported Major Depressive Disorder Diagnosis

Depression was measured by a screening question: “Have you ever been diagnosed with any of the following psychiatric disorders?” Major Depressive Disorder (MDD) is one of the options. The response to this question is either “yes” or “no.” If participants selected “yes” for MDD, they were considered part of the depression group for this study, and vice versa. In our sample, 40% of the participants reported a diagnosis of MDD. In the following sections, we will refer to this as *self-reported depression diagnosis*.

#### Zung Self-rated Depression Scale

The Zung self-rated depression scale consists of 20 items rated on a Likert scale of 1–4 (a little of the time, some of the time, good part of the time, most of the time) that measure the four common characteristics of depression: the pervasive effect, the physiological equivalents, other disturbances, and psychomotor activities [58]. The scores range from 25 to 100, with 50 as clinical depression cutoff. In the following sections, we will refer to this as *self-rated depression symptoms*.

### Social interaction survey and task

#### Social Relations Survey

The Social Relations Survey was adapted from Quality of Life Interview developed by Lehman (1983) [26], which has two subscales. One subscale measures *objective social contacts* with items such as how often you do things with a close friend, visit someone who does not live with you, or call or text someone who does not live with you. The other subscale measures *subjective satisfaction with social relations*, including questions like: How do you feel about the things you do with other people? Are you satisfied with the amount of time you spend with other people? How do you feel about the people you see socially, the number of friendships in your life, and how you get along with other people in general?

#### Social interaction task

**Fig. 1.**
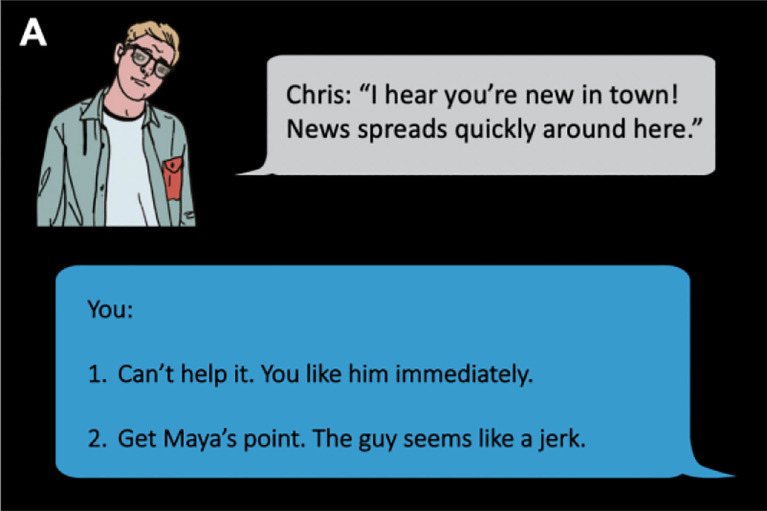
Schematic depiction of a social navigation task trial. The example shows an affiliation interaction. The story line introduces 5 active characters, each with 6 affiliation interactions, providing participants the opportunity to affiliate with the characters.

In this study, participants engaged with a role-playing game designed to map out their individual social landscapes, tracing their interactive pathways with various characters [59]. The game simulated a scenario in which participants find themselves in a new town, with the objective of securing employment and housing by interacting with local residents. During gameplay, participants encountered local residents as cartoon characters on slides, with conversation occurring via text bubbles. These characters lacked a visual spatial context but possessed distinct attributes suggestive of their social roles—such as an old acquaintance or a potential employer. Participants navigated the social scenarios by choosing from two dialogue options, using a button press to dictate their responses. Some of these responses represent social affiliation behaviors with one option representing higher affiliation behavior (= +1) and the other option representing lower affiliation behavior (= −1). An example is shown in Figure 1. This interactive format allows participants to experience a consistent narrative while their choices actively influence the direction of the story, akin to “choose-your-own-adventure” games. We have 5 characters in the game, and for each character, there are 6 trials of affiliation choices. In the analysis, we calculated the mean of affiliation choices across all 30 trials to represent *task affiliation*. After completing the task, participants were asked to rate friendship or intimacy they felt with each character. We then calculated the mean of the affiliation perceptions across the 5 characters to represent *perceived affiliation*.

### Imaging acquisition and processing

Structural MRI data was acquired for all participants on a 7 Tesla whole body scanner (Magnetom, Siemens Healthcare, Erlangen, Germany). A SC72CD gradient coil was used with a single coil transmit and a 32-channel head coil (Nova Medical, Wilmington, MA, USA). A T1-weighted MP2RAGE sequence was performed on each participant, with a 0.7 mm × 0.7 mm × 0.7 mm voxel resolution. Field of view (FOV) was 225 × 183, orientation of scan was coronal, repetition time (TR) was 6000 ms and echo time (TE) was 3.62 ms. A coronal-oblique T2-weighted turbo spin echo (T2-TSE) sequence was also obtained for all participants, with a 0.43 mm × 0.43 mm × 2.0 mm voxel resolution. FOV was 222 × 177, orientation of scan was coronal, TR was 9000 ms and TE was 69 ms. Results included in this manuscript come from preprocessing performed using *fMRIPrep* 22.0.0 (RRID:SCR_016216) [60,61], which is based on Nipype 1.8.3 (RRID:SCR_002502) [62,63]. One T1-weighted (T1w) image was found within the input BIDS dataset. The T1-weighted (T1w) image was corrected for intensity non-uniformity (INU) with N4BiasFieldCorrection [64], distributed with ANTs 2.3.3 (RRID:SCR_004757) [65] , and used as T1w-reference throughout the workflow. The T1w-reference was then skull-stripped with a *Nipype* implementation of the antsBrainExtraction.sh workflow (from ANTs), using OASIS30ANTs as target template. Brain tissue segmentation of cerebrospinal fluid (CSF), white-matter (WM) and gray-matter (GM) was performed on the brain-extracted T1w using fast (FSL 6.0.5.1:57b01774, RRID:SCR_002823) [66]. Brain surfaces were reconstructed using recon-all (FreeSurfer 7.2.0, RRID:SCR_001847) [67], and the brain mask estimated previously was refined with a custom variation of the method to reconcile ANTs-derived and FreeSurfer-derived segmentations of the cortical gray-matter of Mindboggle (RRID:SCR_002438) [68]. Volume-based spatial normalization to two standard spaces (MNI152NLin2009cAsym, MNI152NLin6Asym) was performed through nonlinear registration with antsRegistration (ANTs 2.3.3), using brain-extracted versions of both T1w reference and the T1w template. The following templates were selected for spatial normalization: *ICBM 152 Nonlinear Asymmetrical template version 2009c* [69][RRID:SCR_008796; TemplateFlow ID: MNI152NLin2009cAsym], *FSL’s MNI ICBM 152 non-linear 6th Generation Asymmetric Average Brain Stereotaxic Registration Model* [70][RRID:SCR_002823; TemplateFlow ID: MNI152NLin6Asym]. FreeSurfer (http://surfer.nmr.mgh.harvard.edu) automated segmentation of the volumes was used to extract ACC and amygdala volume. We averaged the volumes of the bilateral rostral anterior cingulate and caudal anterior cingulate to obtain the ACC volume and we averaged the bilateral amygdala volumes as amygdala volume for the subsequent statistical analysis.

### Statistical analyses

The analyses focused on assessing whether autism symptoms and social impairment are related to depression in the ASD population and whether autism- and depression-related brain structures explain this relationship. All *p* values and *CI* were corrected for multiple comparisons within a given question using the false discovery rate.

We ran statistical models to address the following 4 questions using self-reported depression diagnosis first and then repeated the whole procedure on self-rated depression symptoms. To address the first question of whether self-perceived or clinician-endorsed ASD symptoms correlate with depression, we utilized a multiple regression model. This model predicted depression using both clinician-rated ASD symptoms (as indicated by ADOS scores) and self-rated ASD symptoms (as indicated by BAPQ scores), while controlling for age, sex, IQ (previously indicated in the literature as a potential risk factor for depression in ASD), and socioeconomic status.

The second question investigated whether self-reported objective or subjective measures of social interaction are related to depression in individuals with ASD. We applied a multiple regression model to predict depression using measures of both objective social contacts and subjective satisfaction of social relations subscales of the social relations survey, controlling for the same covariates mentioned in the first question. Additionally, we examined whether objective affiliation behaviors during the social interaction task or affiliation perceptions during the task are related to depression in individuals with ASD, again applying the same regression model with both task affiliation and perceived affiliation included.

For our third inquiry into the role of the ACC and amygdala volume in individuals with ASD and depression, we included both brain regions together in our regression models to predict clinician-rated ASD symptoms (i.e., ADOS total scores and ADOS reciprocal social interaction subscale scores) and depression, respectively, controlling for the previously mentioned covariates plus intracranial volume.

Lastly, we sought to determine whether ACC and amygdala volumes correlate with results from subjective ASD symptom, social interaction and affiliation during tasks, using regression models to predict each social interaction outcome with both brain regions included in the analyses. We reported standardized beta coefficients (*β*) and 95% confidence interval (*CI*) to indicate effect sizes in all the analyses along with test statistics and p values.

## RESULTS

### ASD with depression is related to higher subjectively perceived, but not clinician-rated, autism symptoms

**Fig. 2.**
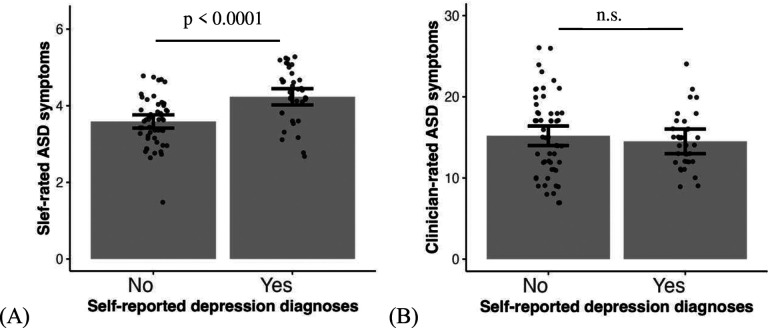

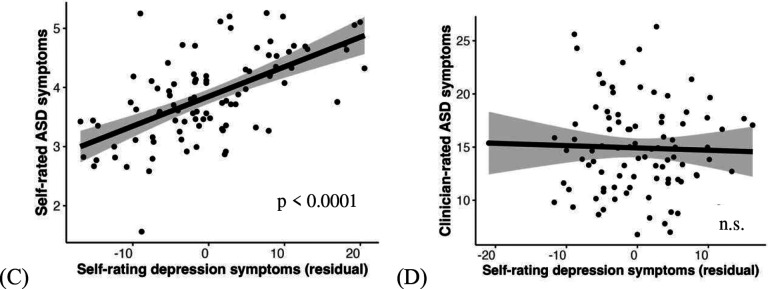
Compared to individuals with ASD without depression, those with ASD and self-reported depression show higher self-rated ASD symptoms (A and C) but no difference in clinician-rated ASD symptoms (B and D). Self-rated depression symptoms in the scatter plots are regressed out covariates including sex, age, SES and IQ.

The first question we examine observed vs subjective measures of ASD symptom in relation to depression. When both self-rated ASD symptoms and clinician-rated ASD symptoms are included together in the model to predict self-reported depression diagnosis, higher self-rated ASD symptoms are associated with a higher chance of depression diagnosis, (*χ*^2^ = 20.36, *β* = 1.28, *95% CI* = [0.52, 2.03], *p* < 0.0001, Fig. 2A), while clinician-rated ASD symptoms are not (*χ*^2^ = 0.32, *β* = −0.15, *95% CI* = [−0.76, 0.46], *p* = 0.892, Fig. 2B). Turning to self-rated depression symptoms, same as self-reported depression diagnosis, higher self-rated ASD symptoms are associated with higher depression symptoms (*F* = 54.82, *β* = 0.64, *95% CI* = [0.44, 0.84], *p* < 0.0001, Fig. 2C), while clinician-rated ASD symptoms are not (*F* = 0.02, *β* = −0.01, *95% CI* = [−0.21, 0.18], *p* = 0.892, Fig. 2D). In our sample, ADOS and BAPQ are not correlated (*r* = −0.08, *p* = 0.448).

### ASD with depression is related to subjective perception but not objective indices of social interaction

**Fig. 3.**
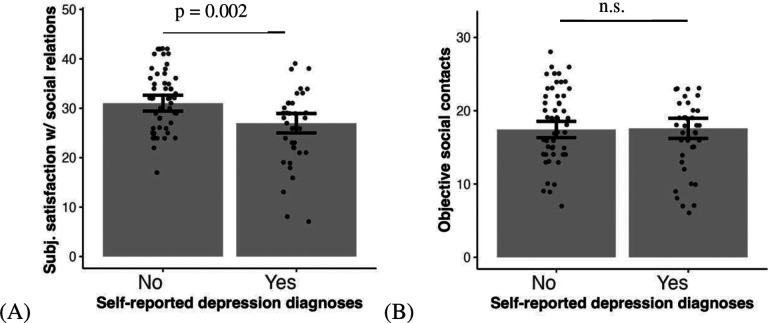

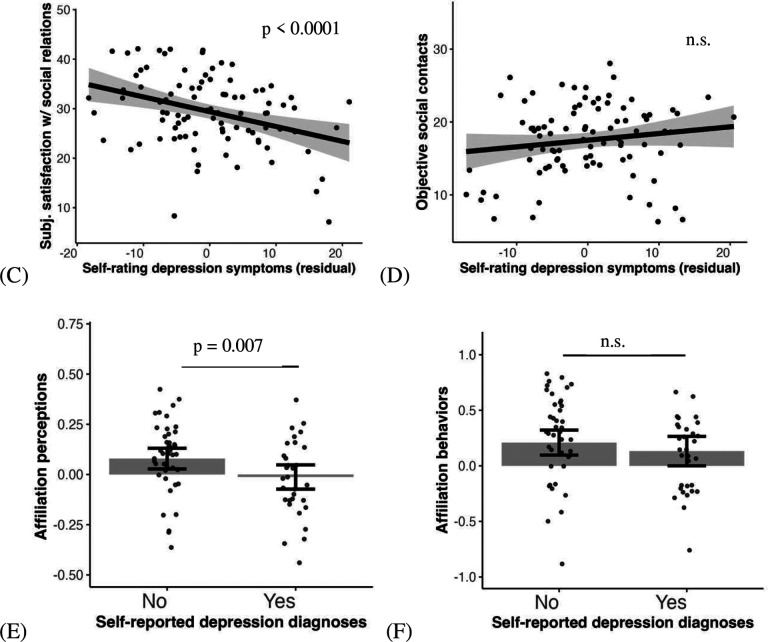
Compared to individuals with ASD without depression, those with ASD and self-reported depression show worse subjective satisfaction of social relations (A and C) and distant perceived affiliation during social interaction (E) but no difference in objective social contacts (B and D) and affiliation behaviors in the task (F). Self-rated depression symptoms in the scatter plots are regressed out covariates including sex, age, SES and IQ.

In addition, when both subscales of the social relations survey (subjective satisfaction with social relations and objective social contacts) are included in the model to predict self-reported depression diagnosis, only lower subjective satisfaction with social relations is related to probability of depression (*χ*^2^ = 9.78, *β* = −1.00, *95% CI* = [−1.80, −0.19], *p* = 0.002, Fig. 3A), while objective social contacts is not (*χ*^2^ = 0.03, *β* = 0.05, *95% CI* = [−0.68, 0.78], *p* = 0.867, Fig. 3B). Likewise, lower subjective satisfaction with social relations is related to higher depression symptoms measured by self-rated depression scale (*F* = 20.96, *β* = −0.54, *95% CI* = [−0.81, −0.27], *p* < 0.0001, Fig. 3C) and objective social contacts are not significant (*F* = 4.34, *β* = 0.26, *95% CI* = [−0.02, 0.54], *p* = 0.080, Fig. 3D).

Finally, when both perceived social affiliation and affiliation behavior in the task are included together in the model to predict self-reported depression diagnosis, only lower perceived social affiliation is related to probability of depression, (*χ*^2^ = 8.45, *β* = −1.03, *95% CI* = [−1.92, −0.14], *p* = 0.007, Fig. 3E), while affiliation behavior is not (*χ*^2^ = 0.55, *β* = −0.21, *95% CI* = [−0.87, 0.44], *p* = 0.460, Fig. 3F). Self-rated depression symptoms are not related to affiliation.

### ASD with depression is associated with larger ACC volume but not associated with amygdala volume

**Fig. 4.**
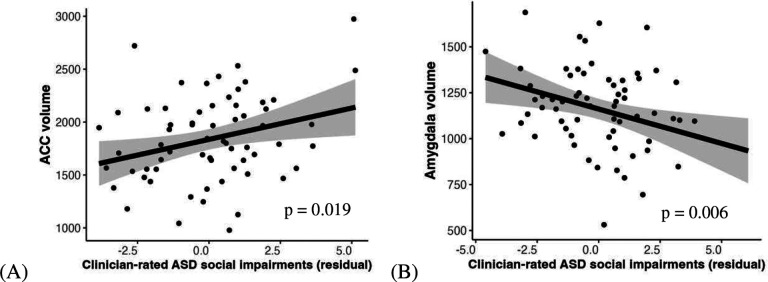
Larger ACC volumes and smaller amygdala and are related to severe social interaction impairment as rated by clinicians (i.e., ADOS reciprocal social interaction subscale). Scatter plots regressed out covariates including sex, age, SES, IQ and intracranial volume.

We examined the regions of interest in the brain in relation to the objective ASD measure (i.e., clinician-rated symptoms) and depression. First, we found that only smaller amygdala volume is correlated with severity of total ASD symptoms endorsed by clinicians (i.e., ADOS total score, for ACC: *F* = 0.18, *β* = 0.35, *95% CI* = [−0.11, 0.48], *p* = 0.148; for amygdala: *F* = 9.59, *β* = −0.39, *95% CI* = [−0.67, −0.10], *p* = 0.006). However, smaller amygdala volume and larger ACC volume correlate with more severe social symptoms as identified by clinicians (i.e., reciprocal social interaction subscale of the ADOS, for ACC: *F* = 7.12, *β* = 0.35, *95% CI* = [0.05, 0.66], *p* = 0.019, Fig. 4A; for amygdala: *F* = 8.29, *β* = −0.37, *95% CI* = [−0.67, −0.07], *p* = 0.006, Fig. 4B). These findings imply that these two regions are relevant to observable features of social impairment associated with ASD.

**Fig. 5.**
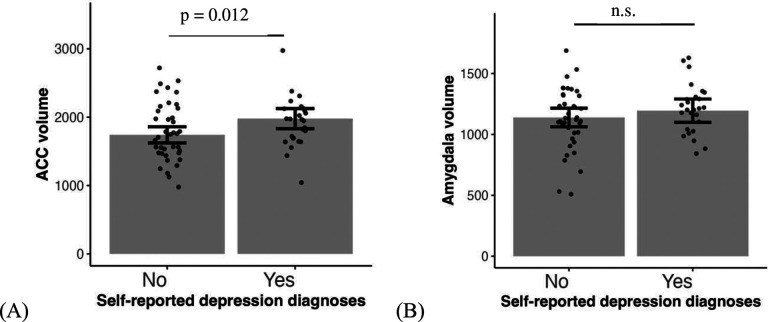

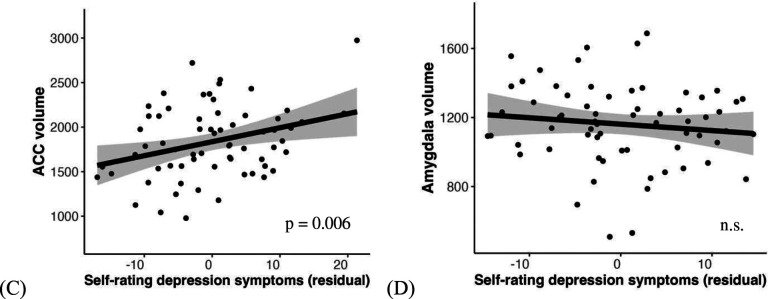
Compared to individuals with ASD without depression, those with ASD and self-reported depression show larger ACC volume (A and C) but no difference in amygdala volume (B and D). Self-rated depression symptoms in the scatter plots are regressed out covariates including sex, age, SES, IQ and intracranial volume.

Next, we analyzed how these two ASD-relevant brain structures are related to depression in our ASD sample. We found that larger ACC volume is associated with higher chance of self-reported depression diagnosis (*χ*^2^= 6.33, *β* = 0.89, *95% CI* = [0.00, 1.78], *p* = 0.012, Fig. 5A). Amygdala volume, however, was not predictive of a depression diagnosis (*χ*^2^= 0.76, *β* = 0.28, *95% CI* = [−0.47, 1.03], *p* = 0.384, Fig. 5B). Similarly, larger ACC volume is also associated with higher self-rated depression symptoms (*F* = 9.57, *β* = 0.41, *95% CI* = [0.10, 0.71], *p* = 0.006, Fig. 5C) but amygdala volume does not show a significant relationship (*F* = 0.76, *β* = −0.12, *95% CI* = [−0.41, 0.17], *p* = 0.384, Fig. 5D).

### Larger ACC volume is related to greater self-perceived ASD symptoms and worse subjective satisfaction of social relations

**Fig. 6.**
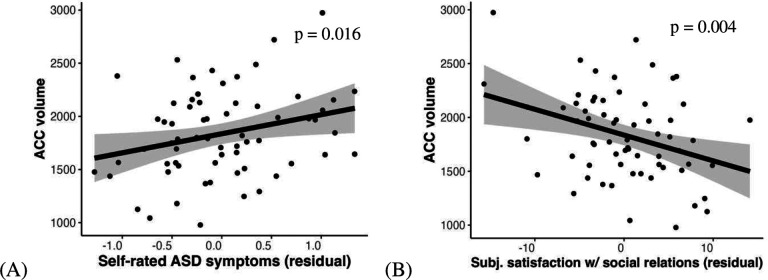
Larger ACC volume is related self-rated ASD symptoms (A) and subjective satisfaction of social relations (B). Self-rated ASD symptoms and subjective satisfaction of social relations in the scatter plots are regressed out covariates including sex, age, SES, IQ and intracranial volume.

Moreover, we assessed the relation of ACC and amygdala volumes to self-rated autistic symptoms and social behaviors. Except that ACC and amygdala volumes are not related to perceived social affiliation, ACC volume is related to self-perceived ASD symptoms (*F* = 6.17, *β* = 0.31, *95% CI* = [0.06, 0.57], p = 0.016, Fig. 6A) and subjective satisfaction with social relations (*F* = 10.35, *β* = −0.36, *95% CI* = [−0.62, −0.10], *p* = 0.004, Fig. 6B). Amygdala volume was not a significant predictor in the analyses, which all showed nonsignificant results (self-rated ASD symptoms: *F* = 0.01, *β* = −0.01, *95% CI* = [−0.25, 0.23], *p* = 0.926; subjective satisfaction with social relations: *F* = 0.63, *β* = 0.08, *95% CI* = [−0.16, 0.33], *p* = 0.861). These findings suggest that the ACC may be sensitive to capturing subjective social interactions associated with depression in ASD. In contrast, the amygdala, being related to objective symptoms of ASD, is not associated with depression or related subjective measures.

## DISCUSSION

Although a high prevalence of depression is observed in the ASD population, the underlying mechanisms remain unknown. It is unclear whether depression is due to the core ASD symptoms related to social deficits or to individuals’ subjective introspection regarding their social impairment, and what brain structures may contribute to this effect remains elusive. Investigating this question can help us understand why some individuals with ASD develop depression while others do not, potentially revealing both risk and protective factors. Our findings indicate that levels of clinician-rated ASD symptoms, objective social contacts, and affiliation behavior during social interaction tasks did not differ between individuals with ASD who did or did not report having depression nor associate with self-rated depression symptoms at the time of participation. However, compared to individuals with ASD without depression, those ASD individuals with depression exhibited heightened self-perceived autism symptoms, worse satisfaction with social relations, and diminished perceived affiliation with social characters during a social interaction task.

Expanding on the findings from Day et al. (2019) in young adults with ASD and low support needs, which indicated that self-awareness of social impairment is related to depression [24], our study expanded the self-assessment to include three measures: self-rated autism traits, subjective satisfaction with social relations, and perceived affiliation during social interaction task. Contrary to Day et al. (2019) and a few other studies [16,24] that found depression to be associated with less severe autism symptoms as assessed by clinicians as well as few studies that found depression to be associated with severe autism symptoms as assessed by clinicians [12–14], we did not find a relationship between clinician-rated ASD symptoms and depression. Consistent with a few other studies, however, we found higher self-rated autism traits related to depression in our autism sample [12,19,71] . Our sample also showed no relation between clinician-rated and self-reported ASD symptoms. Because our sample is restricted to a group of autistic individuals with average or above average IQ, our null relationships may only reflect the segment of the autism spectrum that we were able to include in our assessments. Nevertheless, our findings might imply both common and distinct mechanisms underlying externally observed ASD symptoms and self-introspection of ASD symptoms.

Using structural neuroimaging, we were able to uniquely dissect these common and distinct mechanisms. Interestingly, a larger ACC volume, which is associated with the severity of ASD symptoms of social impairment rated by clinicians and participants’ self-awareness of ASD symptoms, also correlates with a higher likelihood of self-reported depression diagnosis, greater self-rated depression symptoms and worse subjective satisfaction with social relations. On the other hand, amygdala volume relates only to clinician-rated autism symptom severity, but is not related to introspection of autistic traits, depression, or self-assessment of social behaviors. The amygdala has been an important region studied in autism domain, as its association with emotional and social processing [41,72,73]. While some studies found that larger amygdala was associated with more severe ASD symptoms [45–47], we found, consistent with a few other studies [43,44], including one on a male adult sample with average or above average IQ [43], that reduced amygdala volume is associated with more severe ASD symptoms endorsed by clinician. Therefore, despite amygdala volume being not related to depression, it serves as a unique control region in examining the role of the ACC on depression and social impairment in ASD.

Prior studies reported ASD symptom severity in association with ACC functional abnormalities in both social-related tasks [30–32] or resting-state functional connectivity [29]. When investigating ACC structure, one study found that individuals with thinner ACC tended (p < 0.1) to have lower social responsiveness [34] and another study found that reduced white matter volume was related to more social awareness deficits [35]. We contribute to the literature by demonstrating a significant relationship between ACC volume and social reciprocity as rated by clinicians. Moreover, we specifically showed that larger ACC is related to subjective measures including self-awareness of ASD symptoms, self-reported depression diagnoses, and self-rated depression symptom severity, and subjective satisfaction of social relations. As the ACC plays a role in estimating and updating social motivation when interacting with others [28], the ACC might serve as a hub connecting depression with subjective measures of ASD and social satisfaction. It may reflect self-introspection and awareness of autistic symptoms, suggesting that the sources of social satisfaction and depression symptoms are interconnected.

In summary, both the ACC and amygdala volume are related to core ASD symptoms, particularly clinician-rated reciprocal social interaction. However, only the ACC is associated with depression. This suggests that depression might stem from ASD but differs in ways related to the ACC and self-awareness. ACC structure can potentially reveal subgroups in ASD [74] and our findings might suggest subgroups related to depression within the ASD population. One subgroup with enlarged ACC volume may show heightened awareness of social difficulties and a higher risk for depression, while another subgroup with smaller ACC volume may not be aware of their social difficulties, potentially serving as a protective factor against depression. Our findings imply that certain brain regions or networks may be related to ASD-related objective measures but not to subjective perception or depression, and vice versa. Future studies can examine neural pattern-based ASD subtypes based on our findings to differentiate the neural substrates of objective versus subjective manifestations of social interaction and examine their relations to depression.

Our findings are readily translatable for future studies in longitudinal samples. Here, we propose a few future directions based on our findings. Future studies can examine the association between progression of depression symptoms and changes in self-rated ASD symptoms as well as satisfaction in social relationships in order to determine the directional influences of ASD symptoms, self-perception and depression. Future studies could also examine longitudinal tracking of ACC volume during childhood and adolescence, and closely monitor ACC volume to investigate intervention mechanisms and preventative actions for autistic individuals at high risk of depression. Moreover, feelings of loneliness can be an important mediator of social communication deficits in ASD and in the development of depression (see meta-analysis [75]), future studies can examine how feeling of loneliness and introspection interact in this association. Finally, although research on depression comorbidities in ASD has increased in recent years, little has been done to evaluate potential individual × environment interactions associated with these comorbidities [76]. Our study only considered individual factors. Future research should examine the interaction between individual assessments and environmental stressors, an important risk factor for depression across populations, in the context of comorbid depression in ASD (for full reviews, see [17,77,78]).

## LIMITATION

This study had several limitations. As previously mentioned, it is cross-sectional, longitudinal studies are necessary to determine the directionality of the relationships we observe. For example, we found a relationship between self-rated ASD symptoms and subjective satisfaction with social relations and depression in ASD, but we cannot determine whether this self-perception preceded the development of depression or vice versa. Longitudinal studies are needed to determine whether self-assessment is a risk factor for later onset of depression or a marker of current depression. Additionally, the study did not include individuals with an IQ below 60 and required participants to verbally express their experiences interacting with social task characters and self-report on depression and social interaction. These requirements exclude many individuals on the spectrum. Therefore, other risk factors for co-occurring depression should be explored for these groups. Furthermore, we utilized self-reported depression indices instead of clinical diagnosed MDD status. There is some evidence showing that presentation of depressive symptoms may be different in individual with ASD compared with TD individuals, such as more insomnia or restlessness [79–83]. However, there is lack of investigation of the consistent pattern in the self-reports and clinician endorse MDD in ASD [81]. Given rates of depression is much higher if required participants to report on their own depressive symptoms [3], our current study focused on self-report of depression. Future study should incorporate depression measures from both self-report and clinician diagnosis and both current symptoms and past episodes to provide a comprehensive view of relation between depression and ASD.

## CONCLUSIONS

In summary, our findings suggest that higher self-perceived social interaction and enlarged ACC may serve as a potential clinical marker indicating the risk for depressive disorders in ASD. Given the link between depressive disorders, reduced adaptive function, and increased suicide risk, understanding the presentation of depression in ASD may yield significant public health benefits, including improvements in psychosocial outcomes and adaptive functioning. Clinicians can use these features—more self-reported social impairments—to identify individuals with ASD who are at risk for developing depressive disorders. This will also benefit the characterization and diagnosis of ASD with depression and aid in identifying effective treatments.

## Figures and Tables

**Table 1.** Descriptive statistics of demographic and clinical symptoms in study participants (N = 92). Welch two-sample t-tests were performed to evaluate group differences based on self-reported depression diagnoses.

	Self-reported depression Dx		
	YES (37, 40%)	NO (55, 60%)	T-test
Age	28.16 (7.88) 18.17 – 45.78	26.80 (9.89) 18.10 – 56.83	t = 1.08, p = 0.281
Sex	15 males 22 females	34 males 21 females	t = 2.20, p = 0.030
IQ	106.60 (18.78) 67 – 150	104.60 (17.79) 63 – 140	t = 0.82, p = 0.417
SES	−0.09 (1.1) −2.90 – 1.90	0.06 (0.93) −1.89 to 1.90	t = −0.78, p = 0.436
ADOS total	14.54 (3.32) 9 – 24	15.18 (4.86) 7 – 26	t = −0.99, p = 0.325
ADOS Social	8.27 (1.71) 4 – 13	8.35 (2.64) 4 – 16	t = −0.43, p = 0.669
BAPQ	4.27 (0.70) 2.67 – 5.25	3.57 (0.61) 1.56 – 4.69	t = 5.03, p < 0.0001
Zung self-rated depression scale	51.32 (9.72) 35 – 70	43.56 (7.02) 31 – 60	t = 4.60, p < 0.0001

Note: SES (socioeconomic status) is a composite score of the normalized values of income and education.

## Data Availability

The datasets used and/or analyzed during the current study are available from the corresponding author on reasonable request.
